# The ketogenic diet reverses gene expression patterns and reduces reactive oxygen species levels when used as an adjuvant therapy for glioma

**DOI:** 10.1186/1743-7075-7-74

**Published:** 2010-09-10

**Authors:** Phillip Stafford, Mohammed G Abdelwahab, Do Young Kim, Mark C Preul, Jong M Rho, Adrienne C Scheck

**Affiliations:** 1 AZ Biodesign, Center for Innovations in Medicine, Arizona State University School of Life Sciences, Tempe, AZ, USA; 2 Neuro-Oncology Research, Barrow Neurological Institute7 of St. Joseph's Hospital and Medical Center, Phoenix, AZ, 85013, USA; 3 Pediatric Epilepsy Research, Barrow Neurological Institute7 of St. Joseph's Hospital and Medical Center, Phoenix, AZ, 85013, USA; 4 Neurosurgery Research, Barrow Neurological Institute7 of St. Joseph's Hospital and Medical Center, Phoenix, AZ, 85013, USA

## Abstract

**Background:**

Malignant brain tumors affect people of all ages and are the second leading cause of cancer deaths in children. While current treatments are effective and improve survival, there remains a substantial need for more efficacious therapeutic modalities. The ketogenic diet (KD) - a high-fat, low-carbohydrate treatment for medically refractory epilepsy - has been suggested as an alternative strategy to inhibit tumor growth by altering intrinsic metabolism, especially by inducing glycopenia.

**Methods:**

Here, we examined the effects of an experimental KD on a mouse model of glioma, and compared patterns of gene expression in tumors vs. normal brain from animals fed either a KD or a standard diet.

**Results:**

Animals received intracranial injections of bioluminescent GL261-luc cells and tumor growth was followed *in vivo*. KD treatment significantly reduced the rate of tumor growth and prolonged survival. Further, the KD reduced reactive oxygen species (ROS) production in tumor cells. Gene expression profiling demonstrated that the KD induces an overall reversion to expression patterns seen in non-tumor specimens. Notably, genes involved in modulating ROS levels and oxidative stress were altered, including those encoding cyclooxygenase 2, glutathione peroxidases 3 and 7, and periredoxin 4.

**Conclusions:**

Our data demonstrate that the KD improves survivability in our mouse model of glioma, and suggests that the mechanisms accounting for this protective effect likely involve complex alterations in cellular metabolism beyond simply a reduction in glucose.

## Background

Brain tumors will kill ~13,000 people in the US this year, and they are the second leading cause of cancer deaths in children and young adults [1]. Despite currently available treatments, the median survival remains approximately 1 year following diagnosis. Thus, it is of paramount importance that novel and more efficacious therapies be developed for brain cancer patients. One approach is to exploit the metabolic dysregulation seen in tumors which makes them rely preferentially on glucose as an energy source. In support of this concept, the high-fat ketogenic diet (KD) and caloric restriction, both of which reduce blood glucose, have been shown to reduce tumor proliferation in mouse astrocytoma models [2]. Furthermore, two recent case studies [3-5] have suggested that a KD may be a useful therapeutic modality in patients. However, the anti-neoplastic mechanisms underlying such dietary interventions are incompletely understood.

One of the hallmark features of the KD is the increased production of the ketone bodies (i.e., β-hydroxybutyrate [BHB] and acetoacetate [ACA]) which serve as alternative fuels [6], and which have recently been shown to reduce reactive oxygen species (ROS) production in brain [7]. ROS are multi-faceted effector molecules involved in numerous cellular pathways, including those regulating autophagic/apoptotic responses to genotoxic stress, hypoxia and nutrient deprivation. Cancer cells often have increased levels of ROS [8] which have been implicated in angiogenesis induction and tumor growth through the regulation of vascular endothelial growth factor (VEGF) and hypoxia-inducible factor 1 (HIF-1) [9]. In the present study, we examined the effects of an experimental KD in a mouse model of glioma, and found that the KD indeed reduces ROS levels in tumor tissue, and importantly, alters the expression of genes involved in the cellular response to oxidative stress.

## Methods

### GL261 mouse model of glioma

GL261 cells were obtained from DCTD Tumor Repository (NCI, Frederick, MD) and grown in DMEM supplemented with 10% fetal calf serum (FCS) at 37°C with 5% CO_2_. Cells were harvested by trypsinization, washed in DMEM without FCS, resuspended at a concentration of 1-2 × 10^7 ^cells/ml in DMEM without FCS and implanted into female C57BL/6 mice (Jackson Laboratories, Bar Harbor, ME) as described [10]. Each experiment consisted of 20 mice. Mice were propagated in the animal care facility of St. Joseph's Hospital and Medical Center in rooms with controlled temperature and humidity under a 12-hour light-dark cycle. Animals were weighed daily to ensure that all the animals were gaining weight in an equivalent manner. Animals were euthanized at the occurrence of visible symptoms of impending death such as hunched posture, reduced mobility and visible body weight loss [11].

To facilitate a quantitative measurement of tumor growth rate GL261 cells were made bioluminescent using the Lentiphos™ HT System (Clontech Laboratories, Inc., Mountain View, CA) with the Lenti-X™ HT Packaging Mix (Clontech Laboratories, Inc.) and the FUW-GL plasmid (a generous gift from the laboratory of J.B. Rubin, MD, PhD). GL261-luc cells were maintained in DMEM with 10% tetracycline-free FCS (Clontech Laboratories, Inc.). Female C57BL/6-cBrd/cBrd/Cr strain, obtained from the National Cancer Institute Frederick Animal Production Program (Frederick, MD), was used for experiments using GL261-luc cells. Animals received an i.p. or subcutaneous (s.c.) injection of 150 μg luciferin/kg body weight 15 min prior to imaging. *In vivo *imaging was done using an IVIS^® ^Spectrum *in vivo *imaging system (Caliper Life Sciences, Hopkinton, MA). Tumor cells were detectable from the day of implantation and quantitation was done using the system's Living Image^® ^3.1 software.

### Ketogenic diet

Following surgery, animals were fed standard rodent chow for 3 days. Treatment groups were then assigned to remain on regular rodent chow or switch to the ketogenic Bio-Serv F3666 diet (Bio-serv, Frenchtown, NJ) *ad libitum *as described by Rho and coworkers [12,13]. This diet consists of 8.36% protein, .76% carbohydrates and 78.8% fat (173.3 g/Kg casein, 586.4 g/kg cellulose, 586.4 g/kg shortening (Crisco) and vitamins and minerals equal to that found in normal rodent chow). Serum β-hydroxybutyrate levels were determined each week using a Keto-Site reflectance meter (GDS Diagnostics, Elkhart, IN) and blood glucose levels were tested using a HemoCue Glucose 201 System (HemoCue USA, Lake Forest, CA) on blood obtained from tail clips to ensure maintenance of ketonemia and to determine if there was a drop in blood glucose.

### Measurement of Reactive Oxygen Species (ROS

The fluorescent oxidation products of dihydroethidium (DHE, Sigma-Aldrich^®^) were used to demonstrate ROS production *in vivo*, specifically the superoxideo radical. DHE was dissolved in 10% dimethylsulfoxide (DMSO) in PBS and warmed to 40°C. Twenty-seven mg/kg was injected i.p. in a total of 200 μl 18 hours prior to imaging. DHE fluorescence was imaged *in vivo *using an excitation wavelength of 500 nm and an emission wavelength of 620 nm. Spectral unmixing was used to differentiate the DHE signal from autofluorescence. Bioluminescence of the tumor cells was measured as described above.

*In vitro *quantitative ROS measurements were made on cultured GL261 cells and *ex vivo *measurements were made on tumor slices. Cultured cells were treated with a cocktail containing either 2 mM or 10 mM total ketones as described above prior to *in vitro *analysis. *Ex vivo *analysis was done on brain slices containing tumor from animals sacrificed prior to the occurrence of visible symptoms but at least 1 week after the start of the KD. Both cultured cells and brain slices were incubated for 60 min in a standard solution of 145 mM NaCl, 4 mM KCl, 2 mM CaCl_2_, 1 mM MgCl_2_, 10 mM glucose, 10 Mm HEPES, 320-330 mOsm Tris pH 7.4 containing 20 μM 2',7'-dichlorofluorescein diacetate (DCFDA) to measure ROS as described. Cell fluorescence was normalized to background (i.e., cell-free area) and the fluorescence was quantified on an open scale using a value of 0 to represent the absence of detectable signal [7,14].

### Gene expression analysis

Total cellular RNA was isolated from the tumor and the non-tumor containing contralateral side of the brain using the TRIzol^® ^LS Reagent (Invitrogen Corp., Carlsbad, CA) and conditions specified by the manufacturer. Tumor was dissected away from as much non-involved brain as possible prior to extraction. Non-tumor containing contralateral side consisted of the contalateral cortex - an area that did not have tumor cells detectable by histology or bioluminescence. Samples were extensively treated with RNAse-free DNase using the DNA*-free*™ Kit (Ambion, Inc., Austin, TX) and the quality of the sample was ascertained using an Agilent 2100 Bioanalyzer (Agilent Technologies, Palo Alto, CA). Each condition was done in duplicate using two separate animals.

Analysis was performed by the National Institutes of Neurological Disorders and Stroke (NINDS) microarray core facility at the University of California, Los Angeles using the Affymetrix GeneChip^® ^Mouse Genome 430 2.0 array (Affymetrix, Santa Clara, CA). RNA processing and hybridization to the microarrays was performed as recommended by the manufacturer. Hybridized arrays were scanned in a GeneChip Scanner 3000 (Affymetrix) and analyzed with GeneChip Operating Software (GCOS) v1.1 (Affymetrix) was used to perform global scaling to bring the overall intensities of the arrays to a target intensity value of 150. This eliminates biological differences, as well as differences in washings and staining, allowing for inter-array comparisons. The correlation coefficients for the replicate samples were 0.978 for non-tumor samples from animals fed KD, 0.983 for the tumor samples from the animals fed KD, 0.985 for the non-tumor samples from the animals fed SD and 0.993 for the tumor samples from the animals fed SD. Signal values and detection calls were generated in GCOS v1.1 using the Statistical Algorithm, data was imported into GeneSpring 7.3.1 (Agilent Technologies) and each gene was normalized to the median measurement for that gene. Genes showing ≥2-fold difference in expression were used for further analysis.

### Statistical methods

Survival plots used Kaplan-Meier analysis and a Gehan-Breslow-Wilcoxon test. Affymetrix genechip data was analyzed for quality by correlation coefficients across replicates and by measuring the 95^th ^%ile fold-change across technical replicates. The fold-change method is an estimate of the minimal detectable fold-change when only 2 replicates are available [15].

## Results

### The ketogenic diet prolongs survival and inhibits the growth of gliomas *in vivo*

We chose the GL261-C57/BL6 syngeneic intracranial tumor model for this work because it has been used in a number of preclinical therapeutic studies [10,16,17]. This model recapitulates many of the characteristics of human high-grade tumors including invasion, necrosis and pseudopalisading, immunopositivity to GFAP and S100, angiogenesis, high proliferative index, upregulation of vascular endothelial growth factor (VEGF) and hypoxia-inducible factor 1 [alpha] (HIF1[alpha]) around zones of necrosis, mutations in *p53 *and *K-ras*, up regulation of c-myc and p53, and activation of the PI3K pathway as indicated by phosphorylation of Akt [18,19]. The cells have point mutations in the genes encoding *K-ras *and *p53 *and a subpopulation of cells are positive for CD133 and exhibit a stem cell-like phenotype [20].

**Figure 1:**
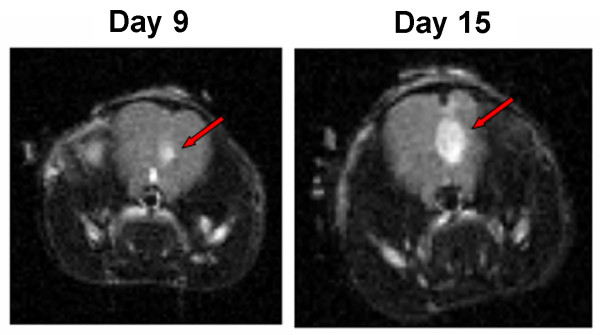
**MRI demonstrates the presence of tumors**. MRI scans were done on tumor-bearing mice on days 9 and 15. Animals were positioned onto a probe in a head first, prone position. A T2 weighted MRI at 4.7 Tesla was obtained on 16 coronal sections of the animal's frontal lobe at a slice thickness of 0.5 mm.

**Figure 2:**
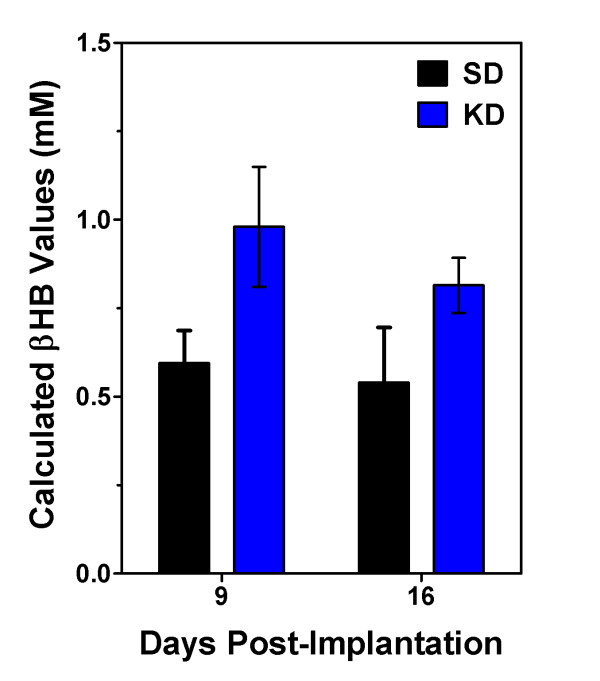
**Animals fed the ketogenic diet showed an increase in blood BHB levels**. There was a statistically significant difference by 2-way ANOVA (p = 0.0067) in blood BHB levels in animals fed KD versus SD. There was no difference in the results obtained at the 2 different time points. Five animals were used per condition and the results are shown as the mean ± SEM.

**Figure 3:**
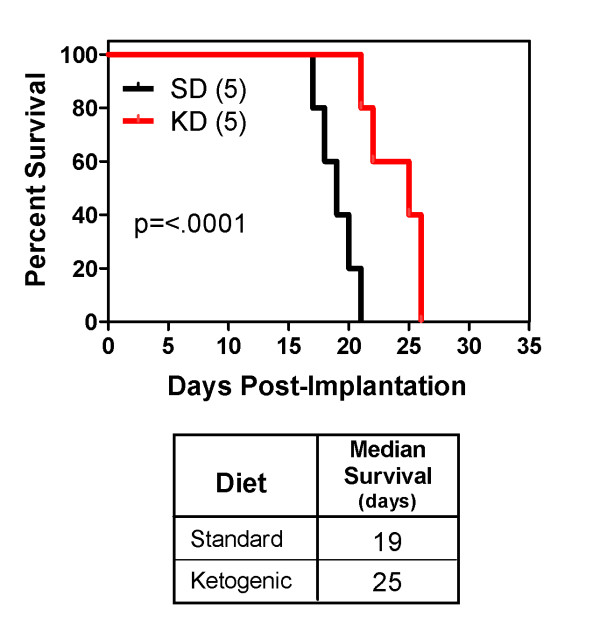
**Animals fed a ketogenic diet had a statistically significant increase in survival following tumor implantation**. The number in parentheses is the number of animals in each treatment group.

MRI scans on days 9 and 15 post-implantation demonstrated tumor growth over time (Figure 1). There was no significant difference in the rate of weight gain between the control animals and the tumor bearing animals regardless of diet SD or KD (data not shown). Animals fed the KD did show a statistically significant (p = 0.0067) increase in BHB levels (Figure 2) as expected. Kaplan-Meier analysis of the survival data using a Gehan-Breslow-Wilcoxon test demonstrated a statistically significant increase (P value of .0196) in survival of animals fed the KD (Figure 3). Tumors were evident in all of the animals on necropsy (data not shown). Brain and tumor tissue was snap frozen in liquid nitrogen for future molecular studies.

**Figure 4:**
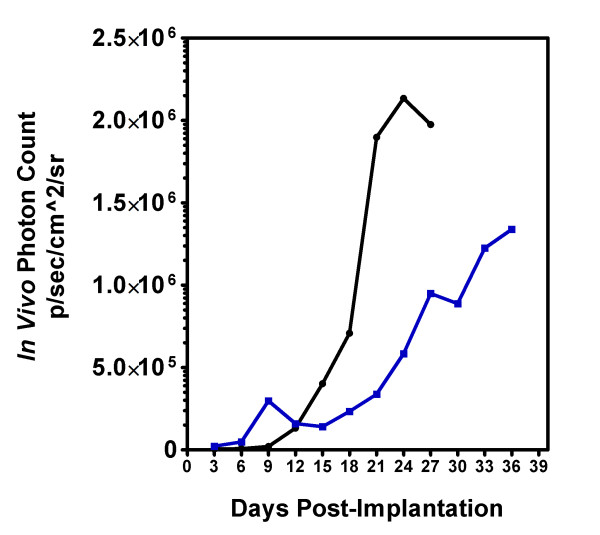
**Tumor growth in animals fed a standard diet (black circle) or a ketogenic diet (blue square)**. Animals were randomized to a treatment arm on day 3 post-implantation. Bioluminescence (photon count) was measured every 3 days. Results are an average of 5 animals for each diet.

The prolonged survival seen in animals fed a KD could be due to a general inhibition of tumor growth, or it could be due to a cytostatic effect of the KD resulting in a lag in tumor growth that was then overcome by the selection of cells resistant to the effects of the diet. The kinetics of tumor growth was demonstrated using GL261-luc cells and *in vivo *imaging. Tumor size was determined every three days following implantation until the animals became symptomatic and were sacrificed. Figure 4 demonstrates that the rate of tumor growth was slowed in animals fed the KD.

### Ketones reduce reactive oxygen species in tumor cells *in vitro *and *in vivo*

**Figure 5:**
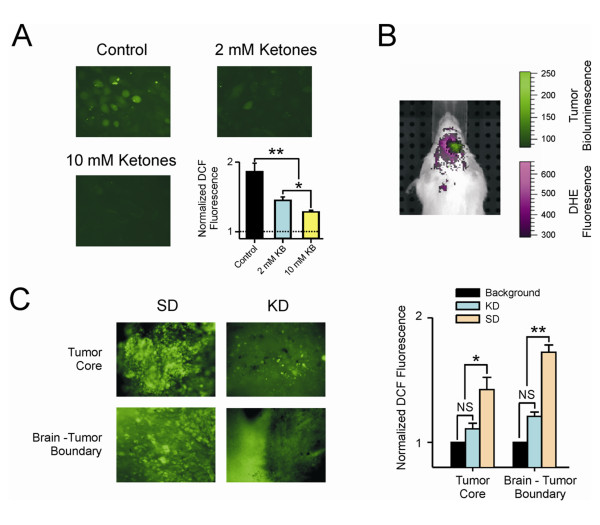
**Ketones reduced ROS levels**. (A) Cells were treated with a cocktail containing either 1 mM (total 2 mM ketones) or 5 mM each (total 10 mM ketones) BHB and AcA for 24 hr prior to analysis. ROS levels were analyzed using DCF as described in the methods. Untreated tumor cells had high levels of ROS, and the application of either 2 mM or 10 mM ketones resulted in a dose dependent statistically significant decrease in the DCF signal demonstrating a reduction of ROS. (* = p ≤ 0.05),(* * = p ≤ 0.001); (B) The presence of increased reactive oxygen species (ROS) in tumor and the surrounding area relative to normal tissue is shown in a mouse fed a standard diet. Dihydroethidium (DHE) was imaged using an excitation wavelength of 500 nm and an emission wavelength of 620 nm. Spectral unmixing was used to differentiate the DHE signal from autofluorescence (shown in purple). The tumor was visualized using bioluminescence (shown in green); (C) Photomicrograph of brain slices from animals fed standard diet (SD) or ketogenic diet (KD). Areas from the tumor core and invading front of the tumor are shown. DCF fluorescence was analyzed as described in the methods section. There is a statistically significant difference in the amount of ROS in tumor vs normal brain when animals fed a standard diet were compared to those fed the ketogenic diet. (* = p ≤ 0.05); N.S. = not significant.

Previous studies in rat neocortical neurons supported the idea that the effect of ketone bodies is primarily through the reduction in ROS [7,14]. To demonstrate the ability of ketones to quantitatively reduce ROS in cultured GL261 cells, we treated them with either 2 mM BHB/ACA or 10 mM BHB/ACA for 24 hr prior to ROS analysis using 20 μM 2', 7'-dichlorofluorescein diacetate (DCF). Tumor cells had high levels of ROS as determined by DCF fluorescence, and the application of either 2 mM or 10 mM ketones resulted in a statistically significant (p ≤ 0.001) decrease in the DCF signal, demonstrating the quantitative reduction of ROS in these cells (Figure 5A).

The presence of increased ROS in the area of the GL261-luc glioma in a mouse maintained on SD was demonstrated using a combination of *in vivo *fluorescent detection of ROS using DHE and bioluminescent detection of the tumor (Figure 5B). While *in vivo *analyses suggested reduced the levels of ROS in animals fed KD (data not shown), it was difficult to obtain a quantitative *in vivo *measure of ROS due to the overlap in emission wavelengths between the DHE and the animals' autofluorescence. We therefore did *ex vivo *analyses on brain slices from animals implanted with GL261-luc cells and sacrificed 2 weeks following implantation. The core of the tumor and the invading front where the tumor intersects with normal brain were analyzed (Figure 5C). There is a statistically significant (p ≤ 0.05) reduction of ROS levels in tumors from animals fed a KD relative to those fed a SD in both the core and the invading front of the tumor. It is of interest that while there is an overall reduction of ROS in tumors from animals fed a KD, we sometimes found cells or small foci of cells that appeared to have a higher level of ROS than the surrounding brain (Figure 5C, arrows). Brain tumors are known to be heterogeneous, and this may represent cells that are resistant to ROS reduction. The data was obtained from 6 cell line experiments and 10 brain slices analyzed using a one-way ANOVA followed by the Tukey test (post-hoc). Data shown is the mean ± SEM.

### Expression profiling analysis

**Figure 6:**
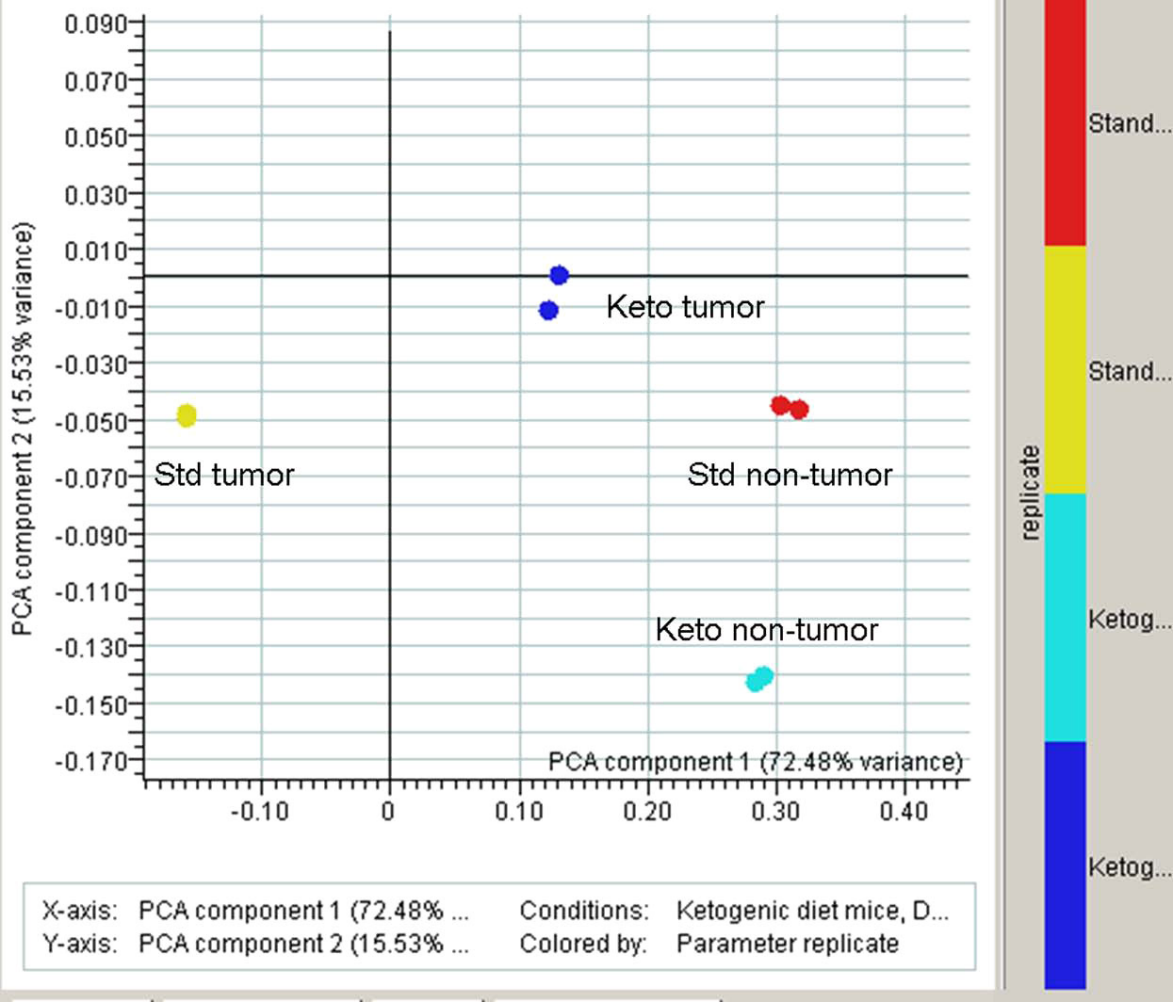
**The KD alters overall gene expression to more closely resemble that seen in normal brain**. Eight microarrays were analyzed by 2-way ANOVA for interaction effects, using standard Bonferonni multiple testing correction. There were strong interactions between ketogenic diet and normal diet in non-tumor classes, especially in context of standard diet non-tumor. The trend is that many expression profiles in tumor mice on a ketogenic diet seem to trend back to a profile seen with mice living on a standard diet having no tumor.

RNA from the tumor and contralateral normal brain was analyzed from 2 animals fed a KD and 2 animals maintained on standard rodent chow. The replicate samples were highly reproducible (≥0.978). A comparison of tumor and normal brain from animals fed a KD demonstrated differential expression of 1129 genes out of the 43,972 transcripts represented on the GeneChip^® ^Mouse Genome 430 2.0 array. Differential expression of 1015 genes was seen in animals maintained on a SD. Of these, 614 genes were common to both gene lists. A two-way ANOVA for interaction was done (Figure 6). The data from the tumor sample obtained from mice fed a SD is clearly separate from the data obtained from the other three conditions. This analysis implies that the KD is driving the overall gene expression in the tumor to be more normal, that is, to be more like gene expression seen in the non-tumor containing tissue.

**Figure 7:**
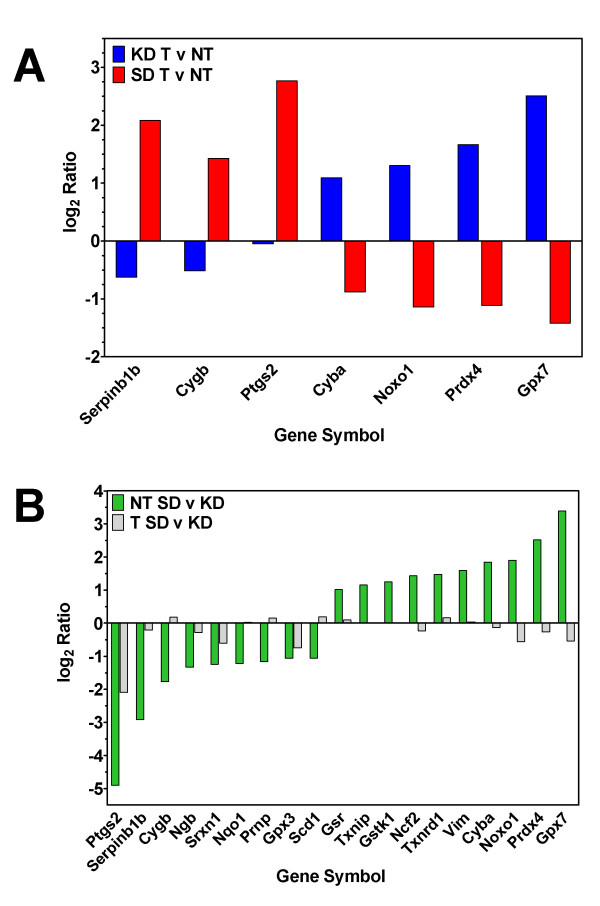
**Expression of genes with significant differential expression in animals fed a KD versus SD**. Log_2 _expression levels of genes that have ≥2-fold expression in at least one condition ratio. 95% confidence intervals are shown.

Since the neuroprotective action of the KD is thought to be due, at least in part, to a reduction in ROS and we demonstrated a reduction in ROS in tumors from animals fed KD, we analyzed the expression of genes involved in ROS metabolism and oxidative stress based on their inclusion in the gene list from the Mouse **"**Oxidative Stress and Antioxidant Defense" RT^2 ^*Profiler *PCR Array from SABiosciences Corp. (Frederick, MD). This 84-gene list includes peroxidases, genes involved in ROS metabolism and oxygen transporter genes. We first analyzed the difference in the ratio of gene expression in tumor versus normal brain from animals fed KD versus SD (Figure 7A, Table 1). A 2-fold difference in expression for at least one of the diets was used as a cutoff, and 9 genes satisfied these criteria. In all cases the effect of the diet was opposite in animals fed SD versus KD. Genes that were over-expressed in tumor versus normal brain in animals fed a SD (*Serpinb1b, Cygb, Mpp4 and Ptgs2*) were under-expressed in tumor from animals fed a KD. Conversely, genes that were under-expressed in tumor versus normal brain in animals fed a SD (*Cyba, Noxo1, Prdx4 and Gpx7*) were over-expressed in tumor from animals fed a KD.

**Table 1:** Genes with significant differential expression in animals fed a KD versus those fed SD.

		SD Expression		KD Expression	
Gene Symbol	Gene Name	Non-Tumor	Tumor	Non-Tumor	Tumor
Ptgs2 (Cox2)	Prostaglandin-endoperoxide synthase 2 (cyclooxygenase 2)	0.732 ± 0.035	0.364 ± 0.01	13.19 ± 5.24	0.41 ± 0.20
Cyga	Cytochrome b-245, alpha polypeptide	5.5 ± 10.86	10.24 ± 0.05	3.79 ± 0.41	13.3 ± 1.31
Cygb	Cytoglobin	8.02 ± 0.06	1.20 ± 0.09	6.53 ± 0.62	1.96 ± 0.01
Gpx3	Glutathione peroxidase 3	9.72 ± 1.33	4.75 ± 0.62	13.16 ± 0.75	5.72 ± 0.54
Gpx7	Glutathione peroxidase 7	2.82 ± 0.14	15.1 ± 0.91	2.18 ± 0.35	23.5 ± 3.07
Gstk1	Glutathione S-transferase kappa 1	8.36 ± 1.13	13.21 ± 1.51	5.83 ± 0.43	13.79 ± 0.56
Ncf2	Neutrophil cytosolic factor 2	1.04 ± 0.02	2.63 ± 0.30	1.31 ± 0.06	3.53 ± 0.25
Ngb	Neuroglobin	3.01 ± 0.02	0.922 ± 0.10	4.06 ± 0.13	1.61 ± 0.01
Noxo1	NADPH oxidase organizer 1	0.56 ± 0.00	1.54 ± 0.31	0.46 ± 0.01	1.54 ± 0.03
Nqo1	NAD(P)H dehydrogenase, quinone	1.75 ± 0.10	0.85 ± 0.03	1.71 ± 0.01	0.744 ± 0.01
Prdx4	Peroxiredoxin 4	2.71 ± 0.21	14.04 ± 0.11	2.48 ± 0.41	19.68 ± 4.01
Prnp	Prion protein	74.09 ± 0.04	21.46 ± 1.43	70.42 ± 9.03	23.17 ± 6.71
Scd1	Stearoyl-Coenzyme A desaturase 1	163.1 ± 5.1	83.65 ± 0.15	163.3 ± 22.16	88.54 ± 4.41
Serpinb1b	Serine (or cysteine) peptidase inhibitor, clade B, member 1b	2.91 ± 0.11	0.39 ± 0.15	3.12 ± 1.53	0.41 ± 0.13
Srxn1	Sulfiredoxin 1 homolog (S. cerevisiae)	0.56 ± 0.00	0.068 ± 0.01	1.17 ± 0.05	0.88 ± 0.02
Txnip	Thioredoxin interacting protein	9.82 ± 1.31	19.66 ± 1.15	8.71 ± 0.46	23.3 ± 1.56
Txnrd1	Thioredoxin reductase 1	15.17 ± 2.54	64.70 ± 1.71	15.69 ± 1.85	46.67 ± 1.33
Vim	Vimentin	15.17 ± 2.23	160.60 ± 3.31	55.58 ± 10.12	140.3 ± 2.24

Expression values are normalized to the 50^th ^percentile per array.

These data suggested that the effect of diet was different in normal brain versus tumor. We therefore compared the effect of diet on normal brain and the effect of the diet on tumor (Figure 7B, Table 1). Twenty-one genes were found to have at least a 2-fold difference in expression in tumor or normal brain from animals fed SD compared to KD. The effect of diet appears to be more pronounced in normal tissue versus tumor tissue. In normal tissue the KD led to the over-expression of 10 genes (*Ptgs2*, *Serpinb1b, Cygb, Ngb, Srxn1, Nqo1, Prnp, Mpp4, Gpx3 and Scd1*) relative to SD. Eleven genes were higher in normal brain from animals fed SD compared with KD (*Gsr, Txnip, Gstk1, Ncf2, Txnrd1, Vim, Cyba, Noxo1, Prdx4 and Gpx7*). Diet had a much less pronounced effect on gene expression in tumor tissue than it did in normal brain. In fact, only one gene (*Ptgs2*) showed more than a 2-fold difference in expression between animals fed SD versus KD.

## Discussion

In the present study, we used a bioluminescent mouse model of malignant glioma to demonstrate that a KD can: (1) significantly retard tumor growth; (2) prevent increases in ROS associated with tumor growth; and (3) shift overall gene expression in tumor tissue to a pattern seen in normal brain. When compared to the expression profile in normal brain, the KD exerts differential effects in brain tumor tissue, and appears to influence specific genes involved in regulation of ROS levels. Taken together, our data suggest that the underlying mechanisms likely involve complex alterations in cellular metabolism beyond a simple reduction in blood glucose, as previously hypothesized [21,22].

The KD is a high-fat, low carbohydrate diet that has been successfully used to treat medically refractory epilepsy for many decades, particularly in children [23]. Intriguingly, recent studies have highlighted potential uses for other neurological disorders [21,24-29]. With respect to brain tumors, Seyfried and co-workers [2,21,30,31] demonstrated that the KD or caloric restriction could extend survival in a mouse model of astrocytoma, and proposed that glucose restriction may be the critical factor, despite a multiplicity of other potential mechanisms [2,21,30-32]. Specifically, while normal brain cells may readily adapt to using ketone bodies as an alternative source of energy, tumor cells are less metabolically flexible. There are myriad reasons for this difference, and changes in gene expression may affect other aspects of glycolysis, respiration and mitochondrial function [21,33,34].

To explore other mechanisms, we used a bioluminescent GL261/C57BL/6 mouse model system in which the KD extends survival in a manner similar to that seen in the CT-A model system used by Seyfried [30,31,35]. Serial *in vivo *imaging of these tumors demonstrated that the KD slowed the overall rate of growth of these tumors, rather than increasing survival by selecting for a subpopulation of cells less influenced by the dietary change. To gain further insight into this observation, we undertook gene expression studies, and compared the profiles to previous studies involving KD treatment in normal rodent brain.

Noh et al [36] studied the hippocampus of normal juvenile mice using a Rat Atlas 1.2 Array II cDNA expression array (Clontech Laboratories) containing 1176 genes. They found 42 genes that were differentially expressed after KD treatment, and interestingly, most encoded proteins are ordinarily involved in mitochondrial metabolic and intracellular signal transduction pathways. Similarly, Bough et al [37] analyzed the effect of a calorie-restricted KD versus SD fed *ad libitum *on gene expression in the hippocampi of normal male rats. They reported up-regulation of genes encoding elements of oxidative phosphorylation and other mitochondrial proteins, as well as some involved in mitochondrial biogenesis. Yet other investigators have studied the expression of genes involved in metabolism using various diets [38], anti-diabetic drugs [39] and genetically altered mice [40].

While there is a dearth of information regarding the mechanisms underlying the putative anti-neoplastic effects of the KD, a number of studies have provided insights into the neuroprotective properties of the diet, and in particular, the role that ROS plays [7,13,41]. This observation was of interest to us since ROS are known to be effector molecules involved in numerous intracellular pathways, including those regulating cellular autophagic/apoptotic responses to genotoxic stress, hypoxia and nutrient deprivation [9,42-46]. Additionally, increased levels of ROS [8] can lead to induction of angiogenesis and tumor growth through regulation of vascular endothelial growth factor (VEGF) and hypoxia-inducible factor 1 (HIF-1) [9]. Several signal transduction cascades activated by tyrosine kinase receptors act in part through ROS-dependent mechanisms [8], and Akt activation by ROS may support tumor cell survival under hypoxic conditions [47]. Finally, ROS may even contribute to the heterogeneity seen in brain tumors because of its differential effects in normoxic vs. hypoxic areas of a tumor [8].

We found that our GL261 cells did indeed exhibit high ROS levels, and when maintained *in vitro*, responded with a significant reduction in ROS upon addition of ketones (Figure 5). Consistently, *in vivo *imaging demonstrated increased ROS in tumor tissue from animals fed a SD (Figure 5B) and *ex vivo *analysis demonstrated the significant reduction of ROS in tumor tissue from animals fed a KD (Figure 5C). While the majority of the tumor showed ROS levels consistent with normal brain in animals fed a KD, it was of interest that a few cells appeared to maintain higher levels of ROS. This may be a reflection of the heterogeneity seen in these tumors. However, it is unlikely that these cells are clinically relevant because no increases in ROS-positive cells were observed over time. Thus, despite the presence of these cells, the many functions of ROS suggest possible pathways through which the KD may affect tumor growth other than alterations in glucose availability.

Given the pivotal role that ROS seemed to play in tumors as a response to the KD, we focused our analysis of gene expression patterns on specific genes involved in modulation of oxidative stress and antioxidant defense pathways. Our statistical analysis revealed that the KD did not substantially alter gene expression in normal mouse brain; however, overall expression in tumor tissue was affected such that it resembled that seen in normal brain issue (Figure 6). Moreover, genes that were over-expressed in tumor relative to non-tumor tissue in animals fed a SD were under-expressed in tumor relative to normal tissue in animals fed a KD (Figure 7B). When we independently analyzed the effect of diet on tumor vs. normal tissue, it was evident that non-tumor tissue generally had a much more robust response to diet than did tumor tissue (Figure 7A).

One prominent gene in our analyses is *Ptgs2 *(*Cox2*). We found that Cox2 expression was reduced to non-tumor levels when animals are fed a KD (Figure 7B). This was particularly intriguing as *Cox2 *inhibition is being explored as a treatment strategy for brain tumors [48-51]. Inhibition of *Cox2 *has been correlated with increased apoptosis in some systems and has been associated with decreased endothelial cell spreading, migration and angiogenesis [48-52]. These data are consistent with a previous report that the KD increased apoptosis and inhibited angiogenesis [30].

Other genes involved in oxidative stress responses were also affected by the KD, including *glutathione peroxidase 7 *(*Gpx7*) and *peroxiredoxin 4 *(*Prdx4*), both of which play cellular protective roles [53,54], and are similarly regulated as Cox2 - i.e., showing higher expression in tumor vs. normal tissue when animals are fed a KD, but not when they are fed a SD. Ziegler et al [55] reported an increase in the activity of the Gpx enzyme in the hippocampus of normal rats fed a KD, but not in the cortex of cerebellum. This study did not examine gene expression profiles, nor did it separate out the isoforms of the Gpx enzyme.

In contrast to *Gpx7 *and *Prdx4*, *cytoglobin *(*Cygb*) - which also protects cells against oxidative stress [56] - was more highly expressed in tumor vs. non-tumor tissue in animals fed a SD, as well as in normal brain when mice were fed a KD. *Cytoglobin *also encodes a tumor suppressor protein [57], and this may help explain why its expression is higher in tumor tissue from animals fed SD, but lower in tumor tissue from animals fed a KD.

Yet another potentially significant player is *Nox organizing protein 1 *(*NOXO1*) which is required for ROS generation by Nox1, along with the *NOX activating protein 1 *(*NOXA1*) [58]. It is not clear why the expression of this gene is higher in tumor than in normal brain in animals fed KD, and why SD-fed animals show higher expression in non-tumor tissue. Adding further complexity is the protein encoded by *cytochrome b245*, *alpha polypeptide *(*Cyba*), which directly interacts with both NOXO1 and NOX1, which in turn leads to the production of H_2_O_2_. The expression of *Cyba *changes with diet and tissue type in a manner identical to the gene encoding NOXO1. Thus, there are a number of genes involved in the formation of ROS through the Nox system, and we cannot rule out the possibility that the KD is less effective at reducing the production of ROS through this mechanism.

Finally, animals fed a KD showed higher expression the gene encoding the serine (or cysteine) peptidase inhibitor, clade B, member 1b (*Serpinb1b*), a putative antioxidant enzyme [59]. This was particularly evident in non-tumor tissue when compared to that seen in animals fed SD; however, *Serpinb1b *expression was higher in KD-fed normal mice, whereas in the tumor groups, animals fed a SD exhibited higher levels. The reason for this difference is not yet clear, but at present, very little is known about the function of this gene product.

## Conclusions

We have shown that the KD, which induces ketonemia, enhances survival in an animal model of malignant glioma, possibly through a reduction in ROS to levels seen in normal brain. Detailed gene expression analyses have revealed that the KD caused overall gene expression to mirror that seen in non-tumor brain tissue. This phenomenon was underscored when genes involved in oxidative stress responses from the various treatment groups were individually analyzed and compared. Furthermore, the effect of the diet on gene expression was more pronounced in non-tumor tissue than it was in tumor tissue. The differential effects on gene expression in the tumor vs. the normal brain suggest that the KD may not only be useful in the treatment of brain tumors, but may also serve a protective function for normal brain tissue during tumor treatment. Finally, our data indicate that the effect of the KD on tumor growth is not simply due to a reduction in available glucose and is more likely to be due to complex interactions amongst a number of gene networks that regulate important intracellular signaling cascades and cellular homeostatic mechanisms.

## Competing interests

The authors declare that they have no competing interests.

## Authors' contributions

PS performed all did statistical analyses on the microarray data and helped draft the manuscript; MGA performed all animal surgeries and followed animals for survival and tumor growth; DYK performed all *in vitro *and *ex vivo *ROS experiments; MCP oversaw the animal surgeries and MRI data interpretation; JMR provided advice and intellectual input into the implementation of the ketogenic diet and edited the manuscript; ACS oversaw all aspects of the project, analyzed microarray results, drafted the manuscript and obtained the funding for the work. All authors read and approved the final manuscript.
